# A Phosphopantetheinyl Transferase from *Dictyobacter vulcani* sp. W12 Expands the Combinatorial Biosynthetic Toolkit

**DOI:** 10.1021/acsomega.5c02708

**Published:** 2025-08-12

**Authors:** Kenneth K. Hsu, Charlie M. Ferguson, Christina M. McBride, Nicholas B. Mostaghim, Kelsey N. Mabry, Robert Fairman, Yae In Cho, Louise K. Charkoudian

**Affiliations:** 1 Department of Chemistry, 3776 Haverford College, Haverford, Pennsylvania 19041, United States; 2 Department of Biology, 3776 Haverford College, Haverford, Pennsylvania 19041, United States

## Abstract

The value of microbial natural product pathways extends beyond the chemicals they produce, as the enzymes they encode can be harnessed as biocatalysts. Microbial type II polyketide synthases (PKSs) are particularly noteworthy, as these enzyme assemblies produce complex polyaromatic pharmacophores. Combinatorial biosynthesis with type II PKSs has been described as a promising route for accessing never-before-seen bioactive molecules, but this potential is stymied in part by the lack of functionally compatible noncognate proteins across type II PKS systems. Acyl carrier proteins (ACPs) are central to this challenge, as they shuttle reactive intermediates and malonyl building blocks between the other type II PKS domains during biosynthesis. To perform this essential role within PKSs, ACPs must first be activated to their *holo* state via the phosphopantetheinyl transferase (PPTase)-catalyzed installation of a coenzyme A (CoA)-derived phosphopantetheine (Ppant) arm. The installation of the Ppant arm is critical to effectively study and strategically engineer type II PKSs, yet not all ACPs can be activated using conventional PPTases. Here, we report the discovery of a previously unexplored nonactinobacterial PPTase from *Dictyobacter vulcani* sp. W12 (vulcPPT). We explored its compatibility with both native and non-native ACPs, observing that vulcPPT activated all ACPs tested in this study, including a noncognate, nonactinobacterial ACP that was not readily activated by the prototypical broad substrate PPTases AcpS and Sfp. Strategic optimization of phosphopantetheinylation reaction conditions increased the *apo* to *holo* conversion catalyzed by vulcPPT. In addition to identifying a promising new PPTase that is easy to prepare and use, this work establishes a roadmap for further investigation of PPTase compatibility and increases access to functional synthase components for use in combinatorial biosynthesis.

## Graphical abstract

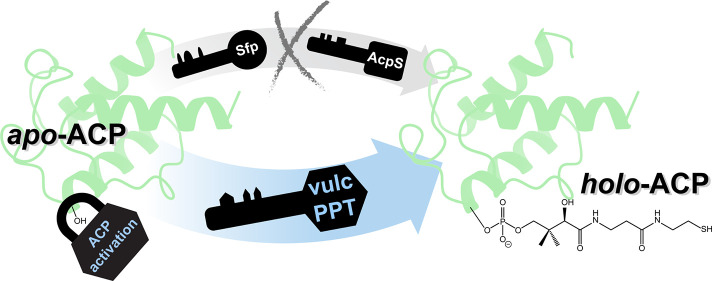

## Introduction

Microorganisms produce a vast array of organic molecules that are not essential to the organism’s survival but confer them with an ecological competitive advantage. Type II polyketides are a particularly exciting class of these secondary metabolites because of their medicinally relevant properties (e.g., the antibiotic tetracycline and anticancer agent doxorubicin).^1^ These molecules are manufactured by multienzyme assemblies known as type II polyketide synthases (PKSs) which are spatially encoded within microbial genomes as biosynthetic gene clusters (BGCs).^1^ At minimum, a type II PKS comprises an acyl carrier protein (ACP), which must be post-translationally modified via the installation of a 4′-phosphopantetheine prosthetic group (Ppant arm) to its active *holo* form, and a ketosynthase-chain length factor (KS-CLF). The activation of the ACP, called phosphopantetheinylation, is catalyzed by an enzyme known as a phosphopantetheinyl transferase (PPTase). The PPTase installs the coenzyme A (CoA)-derived 18-Å long, Ppant arm (Figure 1A) on a conserved serine located on the *N*-terminus of helix II of the ACP.^7^ The Ppant arm enables the ACP to tether molecular building blocks and intermediates throughout the biosynthetic process. It is well-documented that ACPs from PKSs and fatty acid synthases (FASs), as well as peptidyl carrier proteins (PCPs) from nonribosomal peptide synthetases (NRPSs), can be compatible with PPTases encoded by different BGCs within an organism.^8–10^ Similarly, ACPs/PCPs from one organism can be activated by PPTases from different organisms altogether.^11^ The PPTase from the *Escherichia coli* FAS (AcpS)^12^ and the PPTase from the *Bacillus subtilis* surfactin NRPS (Sfp)^13^ and its commonly used R4–4 variant^14^ (referred to as ‘Sfp’ from this point forward), are well-known for their promiscuity in converting noncognate ACPs/PCPs to their *holo* form and are therefore widely used as biosynthetic tools.

**Figure 1:**
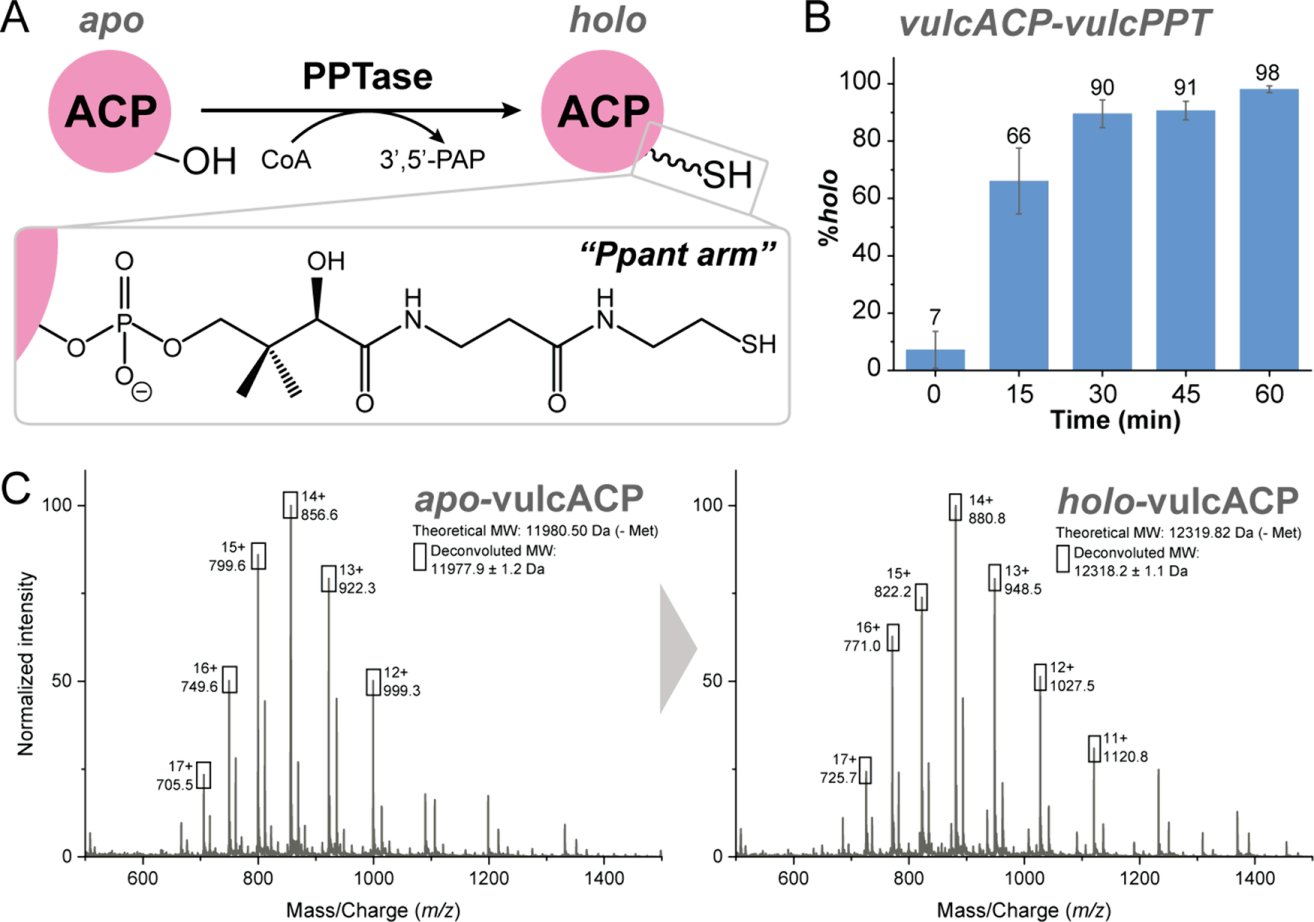
PPTase from *Dictyobacter vulcani* sp. W12 (vulcPPT) converts its putative cognate ACP from the inactive *apo* form to active *holo* form. (A) ACP activation from *apo* to *holo* form is facilitated by a PPTase that installs the 18 Å CoA-derived phosphopantetheine (Ppant) arm on the conserved serine at the *N*-terminus of helix II, releasing 3′5′-phosphoadenosine phosphate (3′5′-PAP). (B) The reaction of vulcACP:vulcPPT over 0 to 60 min at 22 °C (see Materials and Methods for reaction conditions), monitored by LC-MS, shows that vulcPPT efficiently activates its native ACP partner to nearly 100% within 60 min. (C) Mass spectra of *apo*-vulcACP and *holo*-vulcACP (see Figure S5 for more details).

The *holo*-ACP and KS-CLF interact to transform malonyl-based building blocks into a nascent beta-keto polyketide chain through a series of Claisen-like decarboxylation reactions.^1^ Subsequent reactions with a series of accessory enzymes tailor the polyketide intermediate into its final polyaromatic structure. The diversity of type II polyketides originates from variability in both the KS-CLF, which is a primary determinant of the number of carbons in the poly beta-keto chain, and the members of the “tailoring enzyme roster” which catalyze the late-stage biochemical transformation and functionalization of the polyketide backbone.^1^ Unlike type I synthases (or synthetases), each protein or enzyme within a type II PKS exists as a discrete domain, making these systems uniquely conducive to mixing-and-matching components across synthases in an effort to gain access to non-native chemical diversity. However, impaired ACP-protein interactions prevent such combinatorial biosynthesis efforts,^2^ so exploring ACPs that are more suitable for mediation of these interactions is crucial.

ACPs from nonactinobacterial type II PKSs are historically understudied and represent an interesting set of proteins to explore. While recent studies suggest that nonactinobacterial KS-CLFs are uniquely amenable to expression in *Escherichia coli*, their cognate ACPs can be expressed but not activated to their active *holo* form using conventional approaches.^3–6^ For example, the *Photorhabdus luminescens* TT01 type II PKS ACP could not be activated by the *E. coli* PPTase, AcpS, requiring the coexpression of two additional auxiliary enzymes for the successful *in vivo* production of the type II polyketide in *E. coli*.^4^ A similar barrier was encountered in the *in vitro* reconstitution of the *Gloeocapsa sp*. PC7428 type II PKS system in which neither Sfp nor AcpS could convert the *apo*-gloACP, heterologously expressed in *E. coli*, to its *holo* form, requiring strategic mutation of the ACP to enable type II PKS reconstitution *in vitro*.^3^ While the strategic mutation of nonactinobacterial ACPs can confer Sfp compatibility,^3^ identifying new PPTases which can readily activate wild type nonactinobacterial ACPs will improve access to type II PKS components. Herein, we report the *E. coli* heterologous expression and subsequent characterization of a previously unexplored PPTase encoded in the *Dictyobacter vulcani* sp. W12 genome (vulcPPT). VulcPPT demonstrates the ability to activate ACPs that are incompatible with Sfp and AcpS, and therefore represents an important new contribution to the biosynthetic toolkit.

## Results/Discussion

To identify robust PPTases that could readily activate nonactinobacterial ACPs, we turned to previously uncharacterized putative nonactinobacterial type II PKSs BGCs and their associated PPTases^15,16^ for *E. coli* heterologous expression and subsequent characterization. One such type II PKS BGC was identified in the *D. vulcani* sp. W12 genome. Isolated from the soil of the Mt. Zao volcano in Japan, *D. vulcani* sp. W12 is a member of the *Ktedonobacteria* class, which is well-known for actinomycete-like morphology and capability for secondary metabolite production.^17^ Analysis of the *D. vulcani* sp. W12 genome via antiSMASH^18^ revealed a putative type II PKS BGC with a unique, triad-like condensation (KS-CLF) domain architecture^16^ and an ACP with a noncanonical PPTase recognition motif (IDSI instead of LDSL). We selected the sole PPTase (vulcPPT) from the annotated protein list provided in the NCBI whole genome shotgun (WGS) Sequence Set Browser for *D. vulcani* sp. W12 for our heterologous expression efforts, although three additional proteins with homology to *B. subtilis* Sfp and *E. coli* AcpS were identified through BLASTp similarity searches. Interestingly, the genome is predicted to harbor >70 ACPs/PCPs across several putative NRPS and PKS BGCs, suggesting that vulcPPT might display unique and/or broad substrate activity.

The vulcPPT gene (see Materials and Methods and Table S1 in Supporting Information) was cloned into a pET28a-derived construct for *E. coli* heterologous expression with an *N*-terminal His_6_ tag and subsequently purified via affinity column purification to a yield of 60 mg/L. The protein was characterized via SDS-PAGE and liquid chromatography mass spectrometry (LC-MS; Supporting Figures S1 and S2, respectively). The AlphaFold 3-predicted vulcPPT structure depicts vulcPPT as a pseudodimer consisting of two structurally similar subdomains attached by a polypeptide loop with high confidence (Figure S3). The sequence, predicted structure, and size (249 aa) of vulcPPT align with other Sfp-type PPTases.^7^ Circular dichroism (CD) wavelength experiments demonstrate that vulcPPT contains 26.8% α-helical, 8.4% antiparallel β-sheets, and 6.1% parallel β-sheets content.^20,21^ Further protein melting experiments reveal a melting temperature (T_melt_) of 45.53 °C (±0.15 °C) for vulcPPT (Figure S4 and Table S2). Preliminary examination of its *in vitro* activity demonstrates that vulcPPT can fully convert its putative cognate ACP (hereafter referred to as vulcACP) from the inactive *apo* form to active *holo* form within an hour at room temperature (22 °C; Figure 1B and Supporting Information).

To evaluate vulcPPT’s substrate scope relative to PPTases routinely used in the field, we assessed the ability of vulcPPT, AcpS, and Sfp to phosphopantetheinylate four ACPs: (i) vulcACP, (ii) the ACP from the *E. coli* fatty acid synthase (FAS) system (AcpP), (iii) the ACP from the *Streptomyces coelicolor* actinorhodin type II PKS (actACP), and (iv) a previously unexplored ACP from the *Zooshikella* sp. WH53 putative type II PKS BGC (zooACP). These four ACPs were selected to represent diverse substrates, including (i) the putative cognate ACP for vulcPPT, (ii) the prototypical FAS ACP and native substrate for AcpS, (iii) the prototypical type II PKS ACP, and (iv) a never-before-studied noncognate nonactinobacterial ACP. Plasmids encoding the four ACPs were transformed into *E. coli* BL21 (DE3) cells for expression in their majority (∼90–95%) inactive *apo* form. Phosphopantetheinylation reactions were performed by reacting an *apo*-ACP with a PPTase in the presence of CoA, dithiothreitol (DTT), and MgCl_2_ at 22 °C before being quenched at 18 h with formic acid and analyzed via LC-MS. Initial conditions were selected with recognition that overnight reactions at room temperature are easy to implement. Under the LC conditions used (see Materials and Methods for details) the two forms of the ACP (*apo* and *holo*) elute at distinct retention times, allowing for percent conversion to be quantified.

VulcPPT fully converts *apo*-actACP and *apo*-AcpP to their active *holo* states, matching the activity of both AcpS and Sfp (Figure 2B). However, vulcPPT was significantly more efficient in converting its cognate *apo*-vulcACP to its *holo* form than AcpS (100% (±0.0%) versus 58% (±4.5%), respectively). Interestingly, of the three PPTases studied, only vulcPPT was capable of converting zooACP to its *holo* state, albeit at low efficiency (∼11% (±1.5%) *holo*, Figure 2B) under the original conditions evaluated. Together these data highlight the broad substrate scope of vulcPPT and its potential utility in obtaining *holo*-ACPs that cannot be readily obtained using field-standard PPTases. These results are particularly significant in the context of recent work on cyanobacterial PPTases and ACPs, in which diverse PPTases could not outperform Sfp in activating cyanobacterial ACPs/PCPs.^22^

**Figure 2:**
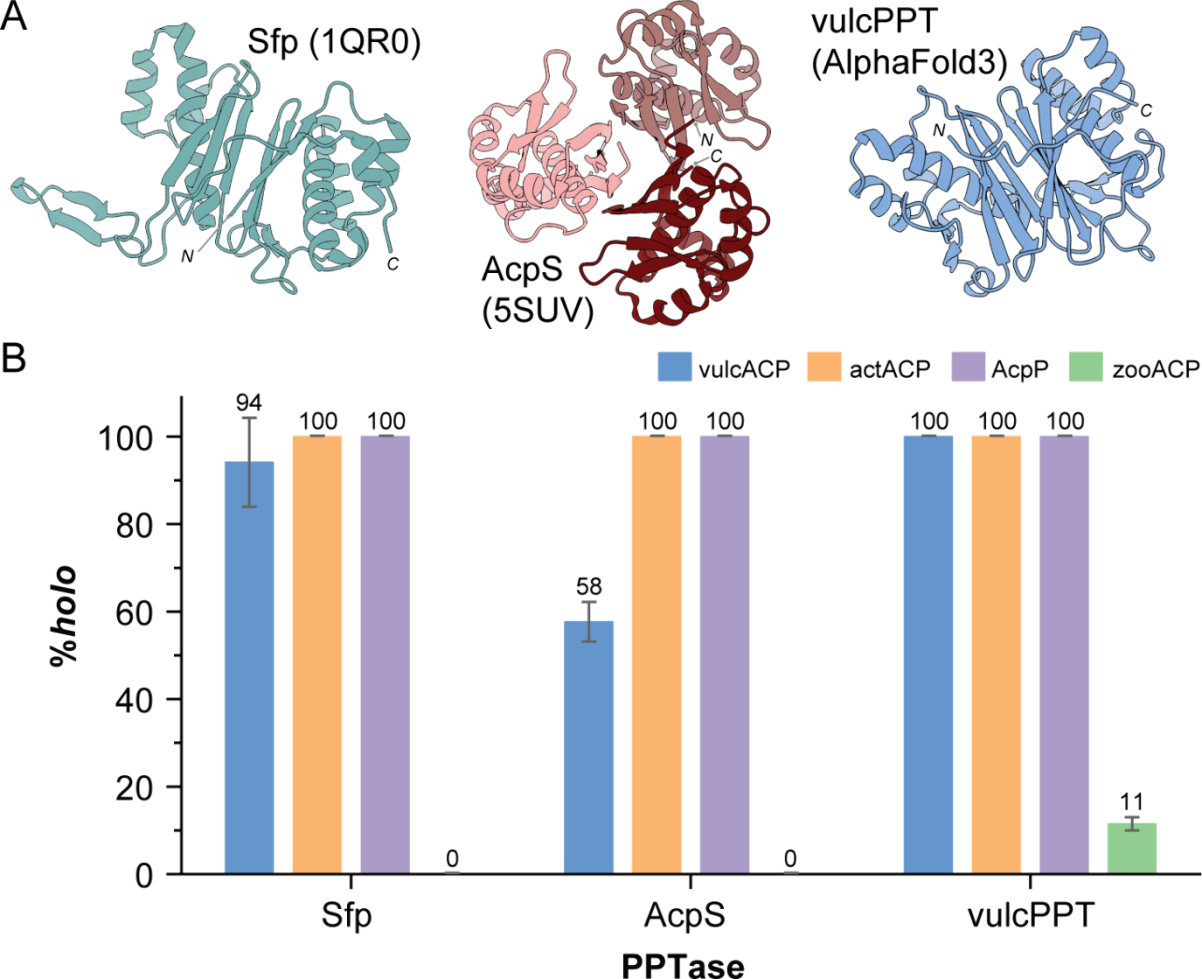
PPTase from *Dictyobacter vulcani* sp. W12 (vulcPPT) shows expanded substrate scope compared to Sfp and AcpS for the ACPs studied at 22 °C. (A) The structures of Sfp (PDB 1QR0), AcpS (PDB 5SUV), and vulcPPT (AlphaFold3 prediction) suggest that vulcPPT belongs to the Sfp-family of PPTases. (B) VulcPPT can activate a range of ACPs, including ACPs that are not readily activated using AcpS and Sfp. When *apo*-vulcACP (blue), *apo*-actACP (orange), *apo*-AcpP (purple), and *apo*-zooACP (green) were reacted with Sfp, AcpS, and vulcPPT (see Materials and Methods for reaction conditions) for 18 h at 22 °C, vulcACP, actACP, and AcpP were converted to their *holo* forms with efficiencies ranging from 58% (±4.5%) to 100% (±0.0%) by all three PPTases. In contrast, zooACP was only activated by vulcPPT, achieving a conversion rate of 11% (±1.5%). See Figures S5–S8 for corresponding LCMS data.

Given promising preliminary results, we next sought to determine whether the *apo* to *holo* conversion efficiency observed for the zooACP:vulcPPT reaction could be improved by altering the phosphopantetheinylation conditions. The four reaction variables—(i) temperature, (ii) pH, (iii) vulcPPT concentration, and (iv) CoA concentration—were assessed independently with 22 °C, pH 7.6, 1.0 μM vulcPPT, and 10x CoA as the standard conditions. First, a 35 °C incubation temperature yielded the highest conversion to *holo-*zooACP (85% (±2.2%)) within the range of 20–37 °C (Figure 3A). Second, vulcPPT produced the highest conversion to *holo*-zooACP (27% (±1.9%)) at pH 7.5, with a broad pH range of activity (Figure 3B). Interestingly, optimal conversion from *apo*-zooACP to *holo*-zooACP was observed at 3.0 μM vulcPPT and 750 μM CoA (Figures S11 and S12, respectively). Taken together, these experiments reveal that the vulcPPT phosphopantetheinylation reaction conditions can be tuned to improve conversion efficiency.

**Figure 3:**
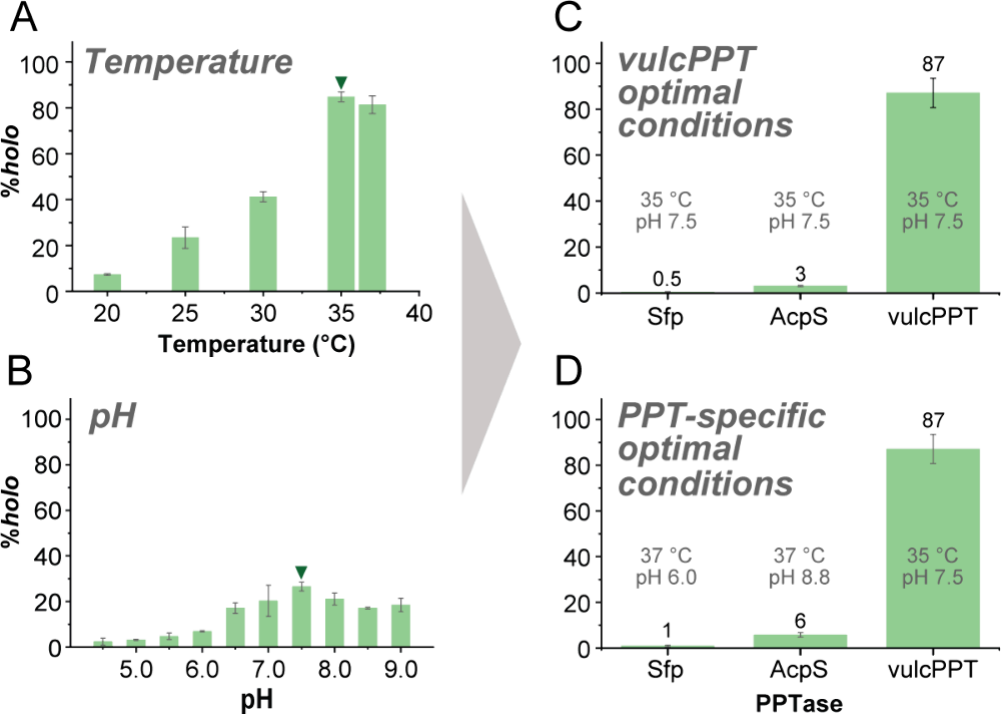
By optimizing reaction conditions, the phosphopantetheinylation of zooACP by vulcPPT could be improved from 11% (±1.5%) to 87% (±6.4%). To optimize the activation of *apo*-zooACP by vulcPPT, different variables were tested: temperature at a constant pH of 7.6 (A, more detailed version in Figure S9), pH at a constant temperature of 22 °C (B, more detailed version in Figure S10), vulcPPT concentration (Figure S11), and CoA concentration (Figure S12). The condition with the highest percent conversion for each condition explored is marked with a triangle. (C) Under the optimized conditions for vulcPPT [35 °C, 750 μM (5 mol equiv relative to *apo*-zooACP = 5x) CoA, 3.0 μM PPT, pH 7.5], *apo*-zooACP was converted to *holo*-zooACP at 87% (±6.4%), which is 8-fold higher than the initial conditions explored. (D) Phosphopantetheinylation of *apo*-zooACP by Sfp, AcpS, and vulcPPT under their respective optimal conditions—pH 6.0 at 37 °C for Sfp, pH 8.8 at 37 °C for AcpS, and pH 7.5 at 35 °C for vulcPPT—followed by analysis after 18 h. While the optimal conditions for vulcPPT were determined in the current study, the optimal conditions for AcpS and Sfp are based on literature reports.^23,24^ The results confirm that only vulcPPT effectively activates zooACP into its *holo* form under these conditions.

Next, vulcPPT phosphopantetheinylation reactions were performed on *apo*-zooACP using the conditions determined to be optimal for each variable explored (3.0 μM vulcPPT, 35 °C, pH 7.5, and 750 μM CoA, Figure 3C). Under these conditions, we observed over 87% (±6.4%) conversion of *apo*-zooACP to its *holo* form. The nearly 8-fold improvement in efficiency compared to the result from unoptimized conditions (11% (±1.5%)) seems to be primarily influenced by temperature (Figure 3A). The zooACP phosphopantetheinylation reaction was performed under identical conditions using Sfp and AcpS in place of vulcPPT which yielded only 0.5% (±0.2%) and 3% (±0.3%) *holo*-zooACP, respectively (Figure 3C). Finally, to further compare the efficiency of all three PPTases, phosphopantetheinylation reactions were conducted on *apo*-zooACP using the literature-reported optimal conditions for Sfp (pH 6.0, 37 °C, Figure 3D) and AcpS (pH 8.8, 37 °C, Figure 3D). For the literature-reported optimal conditions, reactions yielded 1% (±0.4%) and 6% (±1.0%) *holo*-zooACP, respectively—an improvement over the initial reactions using vulcPPT optimal conditions (pH 7.6, 35 °C), yet still much lower than the >87% *holo-*zooACP achieved by vulcPPT. In contrast to what was observed for zooACP, all three PPTases were able to fully convert actACP, AcpP and vulcACP to their *holo* forms at 35 and 37 °C (Figure S13).

We hypothesized that the broad substrate compatibility of vulcPPT is derived from its ability to recognize a broader spectrum of ACP motifs, as compared to Sfp and AcpS.^25^ To gain further insight, the *D. vulcani* sp. W12 genome was analyzed using antiSMASH^18^ to collate a set of putative ACPs from type I/II PKSs and FASs as well as PCPs from NRPSs. 79 ACP/PCPs were identified and further analyzed via multiple sequence alignment (Figure S14). Notably, only a small percentage (6.3%) of these ACP/PCPs contained the traditionally Sfp-favored amino acid motif of DSL (where the S is the conserved serine at the *N*-terminus of helix II that is the attachment point for the Ppant arm). Instead, this DSL motif was frequently replaced by other motifs, such as DSI (44.3%, 35/79) and HSL (34.1%, 27/79) among others. These data suggest that vulcPPT, which is the sole PPTase identified from the annotated protein list in the NCBI WGS Sequence Set Browser for *D. vulcani* sp. W12, may be capable of recognizing a broader range of interaction motifs. This sort of “crosstalk”, where a PPTase activates more than one ACP/PCP in an organism (as opposed to having each PPTase be specific to a single ACP/PCP), has been observed in recent years and can be leveraged in strategic engineering work.^8–11,26^ Additional evidence to support this hypothesis lies in the fact that the threonine in the DST motif has been identified as a key residue that makes an ACP incompatible with Sfp.^3^ Moreover, basal expression of the *E. coli* AcpS could not convert the *Rhizobia* ACP/PCP SMb20651 of unknown function, which contains a DST motif, to its *holo* form. Conversion was only achieved by co-overexpression of the *E. coli* or *S. meliloti* AcpS, suggesting that the presence of a DST motif instead of a DSL motif presents barriers to accessing ACP/PCPs in their *holo* form.^7^ ZooACP features a DST motif and cannot be converted by Sfp to its *holo* form in any meaningful quantity. In comparison, vulcPPT has demonstrated the ability to convert zooACP to its *holo* form in significant quantities, suggesting that vulcPPT is compatible not only with DSL, DSI, and HSL motifs but also with the previously difficult to access DST motif.

The impact of discovering and characterizing new PPTases is highlighted by the ability of vulcPPT to convert *apo*-zooACP, a noncognate ACP which was previously inaccessible using conventional PPTases, to its active *holo* form. It is critical to expand the biosynthetic toolkit such that diverse *holo*-ACPs can be accessed, given that (i) *holo*-ACPs are an essential component to any PKS, whether native or created via combinatorial biosynthesis, and (ii) impaired ACP-protein interactions lead to failure of a PKS to produce a polyketide product. PPTases like vulcPPT allow us to obtain ACPs that are not accessible in their WT form and can currently only be activated by Sfp if the ACP is strategically engineered.^3^ We hope that the introduction of vulcPPT to the combinatorial biosynthetic toolkit will improve access to functional ACPs while also providing additional clues that can be used to uncover the molecular underpinnings of PPTase-ACP compatibility.

## Materials and Methods

### Molecular Cloning of vulcPPT, vulcACP, and zooACP

The plasmids encoding for vulcPPT, vulcACP, and zooACP with *N*-terminal His_6_-tags were created as part of a course-based undergraduate research experience (CURE) in 2023. Template DNAs were purchased from Twist Bioscience, and the primers (both forward and reverse) were purchased from Eurofins Genomics (Table S1). Each template DNA was amplified via polymerase chain reaction (PCR) under the following conditions: 22.5 μL nuclease free water, 10.0 μL 5X Q5 GC enhancer, 10.0 μL 5X Q5 reaction buffer, 2.5 μL forward primer, 2.5 μL reverse primer, 1.0 μL dNTPs, 1.0 μL template DNA, and 0.5 μL of Q5 high-fidelity DNA polymerase for a total volume of 50 μL. In a Bio-Rad S1000 Thermocycler, the reaction mixture underwent a seven-step cycling procedure as follows: 1. 98 °C for 30 s, 2. 98 °C for 10 s, 3. 72 °C for 30 s, 4. 72 °C for 30 s, 5. go to step 2, 29 times, 6. 72 °C for 2 min, 7. hold at 4 °C. Amplification of target DNA was verified via DNA gel electrophoresis (1% (w/v) agarose) and the amplified product was removed and purified using the Zymoclean Gel DNA Recovery Kit. The pET28a vector was linearized through digestion with NdeI and *Eco*RI and purified via DNA gel electrophoresis followed by gel extraction (Zymoclean).

Plasmids were constructed via Gibson Assembly^27^ by inserting the sequence of interest into a pET28a vector at the NdeI and *Eco*RI cut sites. Plasmid inserts and vectors were combined with Gibson Assembly^Ⓡ^ Master Mix (New England BioLabs), consisting of T5 Exonuclease, Phusion Polymerase, Taq Ligase, dNTPs, and MgCl_2_ in Tris-HCl buffer. The reaction mixture was incubated at 50 °C on a heat block. Plasmids encoding for the expression of the actinorhodin ACP (actACP; MC002067), Sfp R4–4, and AcpP were generously provided by the Chang Lab (Princeton University), Lin Lab (Georgia State University), and Khosla Lab (Stanford University), respectively. For details of the constructed plasmids, see Table S1.

### Expression and Purification of ACPs/PPTases

Plasmids were transformed into competent BL21 (DE3) cells for expression. Seed cultures (10 mL Luria-Bertani (LB) supplemented with kanamycin (kan) at 50 μg/mL) were grown at 37 °C and added to production cultures (1 L LB with 50 μg/mL kan). Production cultures were incubated with gentle shaking at 37 °C and induced with isopropyl β-D-1-thiogalactopyranoside (IPTG; 250 μM final concentration) at OD_600_ 0.4–0.6. Induced cultures were incubated with gentle shaking at 18 °C for an additional 12–16 h. Cells were harvested by centrifugation (4424 × *g*, 4 °C, 20 min per round), resuspended in lysis buffer (50 mM sodium phosphate buffer, 10 mM imidazole, 300 mM NaCl, pH 7.6) and sonicated (40% amplitude, 30 s cycles, 10 min) on ice. Lysed cell suspensions were centrifuged (17,000 × *g*, 4 °C, 1 h) and the supernatant was mixed with Ni-NTA agarose beads (Gold Biotechnology) at 4 °C for 1.5–2 h with gentle rocking. The mixture was applied to a poly prep column, and washed with 5 column volumes of lysis buffer, followed by 50 mL of wash buffer (50 mM sodium phosphate, 30 mM imidazole, 300 mM NaCl, pH 7.6) before being eluted with 10 mL of elution buffer (50 mM sodium phosphate, 250 mM imidazole, 100 mM NaCl, 10% (v/v) glycerol, pH 7.6). Ten 1 mL elution fractions were obtained and fractions with A280 values >0.15 were pooled together and dialyzed against a storage buffer (50 mM sodium phosphate, 10% (v/v) glycerol, pH 7.6) overnight. Protein concentrations were determined by Nanodrop 2000 spectrophotometer (Thermo Fisher Scientific). Proteins were flash frozen, and ACPs were stored at 205–437 μM and PPTases were stored at 67–370 μM. Proteins were characterized by SDS-PAGE and LC-MS (see below for details).

### Method to Quantify *apo-*ACP to *holo*-ACP Conversion

Reactions converting *apo*-ACP to *holo*-ACP were routinely run in triplicate using the following protocol unless otherwise specified. For a 125 μL-reaction, *apo*-ACP was incubated with the PPTase in a reaction mixture using stock solutions of 50 mM DTT, 50 mM CoA (lithium salt from CoALA Biosciences), and 1 M MgCl_2_ in 50 mM sodium phosphate buffer, pH 7.6 at 22 °C (final concentrations of each reaction component: 150 μM *apo*-ACP; 1 μM PPTase; 2.5 mM DTT; 1.5 mM CoA; 10 mM MgCl_2_).

To adjust the pH of each reaction mixture, the *apo*-ACP stock in 50 mM sodium phosphate buffer (pH 7.6) was concentrated to 750 μM such that the ACP occupied only 20% of the final reaction volume. The remaining reaction volume was reached by adding 50 mM sodium phosphate buffer of varying pH. All reactions were run for 18 h. To stop the reaction, 50 μL of the reaction mixture was removed and quenched with 10 μL of 25% formic acid for 30 min. The quenched sample was prepared for LC-MS by adding 20 μL of 5% NaOH and 20 μL of LC-MS grade water. All reactions were run in triplicate with averages and standard deviations reported. For more detailed LC-MS methods and the percent quantification of *apo/holo* of these ACP samples, see below.

### Liquid Chromatography–Mass Spectrometry (LC-MS)

An Agilent Technologies InfinityLab G6125B LC/MS coupled with an Agilent 1260 Infinity II LC system equipped with a Waters XBridge Protein BEH C4 reverse phase column (300 Å, 3.55 μm, 2.1 mm × 50 mm) and XBridge Protein BEH C4 Sentry guard cartridge (300 Å, 3.5 μm, 2.1 mm × 10 mm) was used to analyze samples at 45 °C by electrospray ionization mass spectrometry (ESI MS) in positive mode. The instrument was equipped with two LC-MS grade solvents: Solvent A (H_2_O + 0.1% formic acid) and Solvent B (acetonitrile + 0.1% formic acid). The following gradients were used depending on the sample:

- **For the samples not requiring the** *apo/holo* **quantification:** 0–1 min 5% B; 1–3.1 min 95% B; 3.1–4.52 min 95% B; 4.52–4.92 min 5% B; 4.92–9 min 5% B. Unless otherwise stated, LC-MS samples were prepared by diluting 10 μL of protein sample with 90 μL of LC-MS grade water to the approximate concentration of 5–10 μM. Samples were injected (20 μL) and run using a capillary voltage of 3000 V and a fragmentation voltage of 75 V.
- **For the ACP samples to confirm the** *apo/holo* **quantification:** The C4 column was first equilibrated for 10 min at a 0.400 mL/min flow rate with 10% B, after which 20 μL of sample was injected into the column. The following solvent gradient was used post injection: 0–5 min 30% B; 5–30 min 50% B; 30–36 min 95% B; 36–41 min 10% B. Samples were run using a capillary voltage of 3000 V and a fragmentation voltage of 75 V.

Acquired mass spectra were deconvoluted using ESIprot online^28^ and the observed and calculated molecular weights (MWs) were compared to confirm a successful phosphopantetheinylation. LC absorbance data at 280 nm were zeroed and plotted in Origin 2023b. Relative quantities of *apo*- and *holo*-ACP were calculated by integrating the respective UV–vis absorbance of eluent peaks.

### Sodium Dodecyl Sulfate-Polyacrylamide Gel Electrophoresis (SDS-PAGE)

For nonreducing SDS-PAGE samples, 5 μL of 6x purple gel loading dye (New England Biolabs) were combined with 25 μL protein samples (20 μM for ACPs; 15 μM for PPTases). For reducing SDS-PAGE samples, 5 μL of 6x purple gel loading dye and 1.5 μL of β-mercaptoethanol were combined with 23.5 μL protein samples (20 μM for ACPs; 15 μM for PPTases), making 5% (v/v) β-mercaptoethanol overall. Samples were denatured at 100 °C for 5 min and 10 μL of each sample was loaded in each well of the gel (Bio-Rad Mini-PROTEAN^Ⓡ^ TGX 10-well, 30 μL 4–20% precast polyacrylamide gels). SDS-PAGE gels were run in 1X SDS-PAGE running buffer (from the 10X: 0.25 M Tris base, 1.92 M glycine, 1% (w/v) SDS, pH 8.3) at 120 V. Gels were washed with ddH_2_O, stained with Thermo Fisher GelCode Blue for 1 h with gentle shaking, destained overnight with ddH_2_O with gentle shaking, and imaged using a Bio-Techne FluorChem M System (Figure S1).

### Circular Dichroism

CD spectra were collected using an Aviv Model 410A circular dichroism spectropolarimeter. Protein samples were diluted to 200 μL to final concentrations of 10 μM in the storage buffer (50 mM sodium phosphate, 10% (v/v) glycerol, pH 7.6). Samples were injected into a High Precision Quartz SUPRSIL cuvette with 0.1 cm path length (Hellma Analytics). The spectropolarimeter was purged with nitrogen for 2 h. After the UV lamp was warmed up for 30 min, CD spectra were collected at 25 °C with a range of 190–250 nm using bandwidth of 1 nm, a 0.5 nm step size, averaging time of 3 s, and 5 scans. To assess thermal stability, changes in signal at 222 nm were monitored as a function of temperature under the following parameters: 10–90 °C, 2 min equilibration, heating rate 2 °C min^–1^, 30 s signal averaging time, and 1 nm bandwidth. Pre- and postmelting spectra were smoothed using a smoothing function implemented in the Aviv software, using a window width of 11 data points, degree 2. The resulting spectrum (Figure S4) was converted to units of mean residue ellipticity (MRE) using the amino acid sequence of the protein and the sample concentration. Analysis of protein secondary structure characteristics was conducted by uploading normalized data in units of MRE to the web server BeStSel.^20,21^ Normalized data was plotted in Origin 2023b. To calculate the melting temperature of a protein (T_melt_), changes in signal at 222 nm as a function of temperature were plotted in Origin 2023b. A sigmoidal curve was fitted to the plot using a Levenberg–Marquardt algorithm (logistic function) and the E50/x0 value was taken as the T_melt_ of vulcPPT (Figure S4 and Table S2).^29^

## Supplementary Material



## Notes

Plasmid, primer, and amino acid sequence information; SDS-PAGE of the purified ACPs and PPTases; LC-MS spectra of the *apo-* and *holo-*ACPs upon phosphopantetheinylation; CD spectrum and T_melt_ of vulcPPT; temperature, pH, vulcPPT concentration, and coenzyme A concentration optimization of the phosphopantetheinylation of *apo*-zooACP by vulcPPT; multiple sequence alignment of 79 CPs encoded by the *Dictyobacter vulcani* sp. W12 genome (PDF)

## ^

Department of Chemistry and Biochemistry, Providence College, Providence, RI 02918, USA

## ABBREVIATIONS

PKS: polyketide synthase
BGC: biosynthetic gene cluster
ACP: acyl carrier protein
PPTase: phosphopantetheinyl transferase
CoA: coenzyme A
AcpS: *holo*-(acyl carrier protein) synthase
Sfp: 4’-phosphopantetheinyl transferase protein from *Bacillus subtilis*
NRPS: nonribosomal peptide synthetase
KS-CLF: ketosynthase chain length factor
Ppant arm: 4’-phosphopantetheine prosthetic group
SDS-PAGE: sodium dodecyl sulfate polyacrylamide gel electrophoresis
LC-MS: liquid chromatography mass spectrometry
CD: circular dichroism
