# Supplementary material for: A Phosphopantetheinyl Transferase from Dictyobacter vulcani sp. W12 Expands the Combinatorial Biosynthetic Toolkit
Source: ACS Omega. 2025 Aug 12;10(33):37276–83. doi: 10.1021/acsomega.5c02708 (PMC12391972; doi:10.1021/acsomega.5c02708)
Supplement: Supplementary file 1 [file ao5c02708_si_001.pdf]

Supporting Information for:

A Phosphopantetheinyl Transferase from *Dictyobacter vulcani* sp. W12 Expands the Combinatorial Biosynthetic Toolkit

Kenneth K. Hsu,[1] *Charlie M. Ferguson,*[1] *Christina M. McBride,*[1] *Nicholas B. Mostaghim,*[1] *Kelsey N. Mabry,*[1] *Robert Fairman,*[2] *Yae In Cho*[1,*] *and Louise K. Charkoudian*[1,*]

[1]Department of Chemistry, Haverford College, Haverford, PA 19041

[2]Department of Biology, Haverford College, Haverford, PA 19041

*Corresponding authors: Yae In Cho (ycho@providence.edu) and Louise K. Charkoudian (lcharkou@haverford.edu)

Table of Contents **Page**

Table S1. Plasmid, primer, and amino acid sequence of all proteins used in this study. 3

Table S2. Thermal stability curve fit of vulcPPT. 4

Figure S1. SDS-PAGE characterization of the expressed and purified acyl carrier proteins (ACPs) and phosphopantetheinyl transferases (PPTases) used in this study. 5

Figure S2. LC-MS spectrum of purified vulcPPT. 6

Figure S3. Predicted structure of vulcPPT (AlphaFold 3) and its overlay with Sfp. 7

Figure S4. Circular dichroism spectrum and T_{melt} of vulcPPT. 8

Figure S5. Mass spectra of *apo*- and *holo*-vulcACP, and LC chromatograms after PPTases reactions with Sfp, AcpS and vulcPPT. 9

Figure S6. Mass spectra of *apo*- and *holo*-actACP, and LC chromatograms after PPTases reactions with Sfp, AcpS and vulcPPT. 10

Figure S7. Mass spectra of *apo*- and *holo*-AcpP, and LC chromatograms after PPTases reactions with Sfp, AcpS and vulcPPT. 11

Figure S8. Mass spectra of *apo*- and *holo*-zooACP, and LC chromatograms after PPTases reactions with Sfp, AcpS and vulcPPT. 12

Figure S9. Temperature optimization of the phosphopantetheinylation of *apo*-zooACP by vulcPPT (detailed version of Fig 3A). 13

Figure S10. pH optimization of the phosphopantetheinylation of *apo*-zooACP by vulcPPT (detailed version of Fig 3B). 13

Figure S11. VulcPPT concentration optimization of the phosphopantetheinylation of *apo*-zooACP by vulcPPT. 14

Figure S12. Coenzyme A concentration optimization of the phosphopantetheinylation of *apo*-zooACP by vulcPPT. 14

Figure 13. Phosphopantetheinylation reactions of Sfp and AcpS with vulcACP, actACP and AcpP at 35 ºC and 37 ºC. 15

Figure S14. Multiple sequence alignment of 79 carrier proteins encoded by the *Dictyobacter vulcani* sp. W12 genome. 16

References for Supporting Information 17

Tables and Figures

Table S1. Plasmid, primer, and amino acid sequence of all proteins used in this study.

vulcPPT	Forward Primer:<br>CCT GGT GCC GCG CGG CAG CCA TAT GAT CGA AGA CAT CTG GC<br>Reverse Primer:<br>AAG CTT GTC GAC GGA GCT CGA ATT CTC ACA ACG TGC CGT TCC ATT GC<br><br>Insert:<br>ATGATCGAAGACATCTGGCAGCCACCTCCCTCAACATTAAAACTTGAGCAAAGCGCAGTCCAC<br>GTGTGGCGTGCTGACCTTCGTGCCTCTCAAGAATCGGTCGAACGTTTCCGCCACATCCTTTCGC<br>CTGAGGAGCAGGCACGCGCGCAGCGTTTCTACTTCGAACGTGATCGTTACCGTTGGACTATTGC<br>ACATGGCATCTTGCGTATCCTTTTGGCCCGTTACACTGGGCAAGATCCACGCGCCTTGCGCTTT<br>CAAGTCAACGCCTACGGGAAGCCTTCCCTGGTGCAGCCCGATCAACAACCCCGCCTGGAATTT<br>AACCTTAGCCACTCTCATGAGATGGCTTTATATGCTTTTACGTGGCAGCGCCAGATTGGGGTGG<br>ACGTAGAGTACATGCGTGATGATATTGGGTATGAAGAATTGGCTCGCCACTCCTTCAGCCCGA<br>CCGAGCAAGCCGTTCTTCTGTCGTTGGCGACCTCGCAACAAAAAGCCGCTTTCTTTAAATGCTG<br>GAGTTCCAAGGAAGCTTATATCAAGGGTCGCGGGATGGGTCTGTCTTTAGAGCTTAACCTTTTT<br>GACGTGGCTCTGGCTCCCGACAAGCCGGTTGCACTGTTAGCTTCCCGTGAAGACCCAGCCGAA<br>GTCCAACGCTGGTCAATGGCAAAACTGGAACCGGGTGCCGACTACGCTGGTGCGTTGGCGGTC<br>GAAGGGCCTCTTCCGGATATCTCTTGTTGGCAATGGAACGGCACGTTGTGA<br><br>Amino Acid Sequence:<br>MGSSHHHHHHSSGLVPRGSHMIEDIWQPPPSTLKLEQSAVHVWRADLRASQESVERFRHILSPEEQ<br>ARAQRFYFERDRYRWTIAHGILRILLARYTGQDPRALRFQVNAYGKPSLVQPDQQPRLEFNLSHSH<br>EMALYAFTWQRQIGVDVEYMRDDIGYEELARHSFSPTEQAVLLSLATSQQKAAFFKCWSSKEAYI<br>KGRGMGLSLELNLFDVALAPDKPVALLASREDPAEVQRWSMAKLEPGADYAGALAVEGPLPDISC<br>WQWNGTL
vulcACP	Forward Primer:<br>CCT GGT GCC GCG CGG CAG CCA TAT GGT CAA TGA CGC TCT GC<br>Reverse Primer:<br>AAG CTT GTC GAC GGA GCT CGA ATT CTC ACT TGC TGG CGG CTT CC<br><br>Insert:<br>ATGGTCAATGACGCTCTGCAAGAGACGATTTATCCGCGCGTAGTAGCTATTTTACGTTGCCAAG<br>TAGACGAAGACGATGAAATTCGCTCTGATACCGACCTGTTAACAGATTTAGGAATTGACTCAA<br>TCGGCCAAGTCGAAATCTGCCTTGCGCTGGAGAAAGAGTTCGGACTGCGCTTCTCCATTGCAG<br>AGCTTCGCGTTTGCACGACAGTAGATGAGGTGGTTCAGCTTGTCGTACACACGATTGCGGGTA<br>AGGAAGCCGCCAGCAAGTGA<br><br>Amino Acid Sequence:<br>MGSSHHHHHHSSGLVPRGSHMVNDALQETIYPRVVAILRCQVDEDDEIRSDTDLLTDLGIDSIGQVE<br>ICLALEKEFGLRFSIAELRVCTTVDEVVQLVVHTIAGKEAASK

zooACP	Forward Primer:<br>CCT GGT GCC GCG CGG CAG CCA TAT GAC CAA CGA AAC GGT TA<br>Reverse Primer:<br>AAG CTT GTC GAC GGA GCT CGA ATT CCT AAG CGG CCT CGC TTA AA<br><br>Insert:<br>ATGACCAACGAAACGGTTAAGGCCATCTACACAACGCTGGCGGAGTATTTGGATATG<br>CCGGTGTCTGAGTTGAAGGAGAATACAAACTTGGAAAACGAACTTCTGTTGGACTCT<br>ACTGAATTGGTCTGCATTATGGTAGCCTTGGAAAAATCTATGGATATTAGTCTGAAA<br>AATGTGCCTTTCAAGGATTGGGTTGTCATTAACGATATTGTGAATGCAGTGGAGCAG<br>CGTTTAAGCGAGGCCGCTTAG<br><br>Amino Acid Sequence:<br>MGSSHHHHHHSSGLVPRGSHMTNETVKAIYTTLAEYLDMPVSELKENTNLENELLLDST<br>ELVCIMVALEKSMDISLKNVPFKDWVVINDIVNAVEQRLSEAA

Table S2. Thermal stability curve fit of vulcPPT.

Curve Fitting Model	Logistic
Equation	y = A2 + (A1-A2)/(1 + (x/x0)^p)
A1	-8033.27984 ± 40.53121
A2	-962.00728 ± 37.40994
x0	45.52884 ± 0.14873
E50	45.52884 ± 0.14873
p	14.08977 ± 0.56409
Reduced Chi-Sqr	21690.66673
R-Square (COD)	0.99812
Adj. R-Square	0.99797

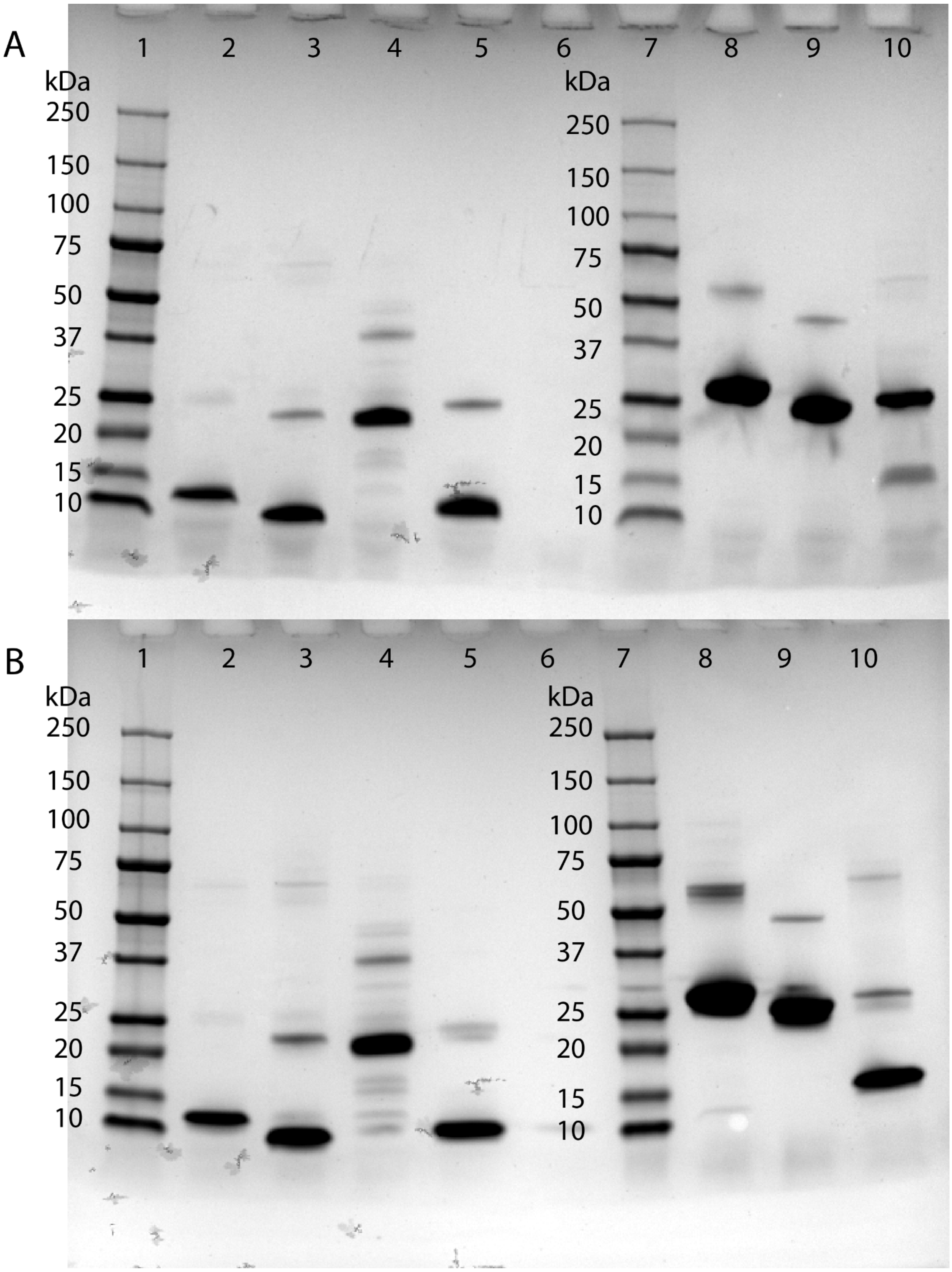

Figure S1. SDS-PAGE of the expressed and purified acyl carrier proteins and phosphopantetheinyl transferases used in this study. Gels were run under (A) non-reducing conditions and (B) reducing conditions with 5% (v/v) β-mercaptoethanol. In both gels, lanes 1–10 represent: (1+7) Precision Plus Protein^{TM} Standards All Blue protein ladder (Bio-Rad); (2) vulcACP (12.1 kDa); (3) actACP (11.4 kDa); (4) AcpP (10.8 kDa); (5) zooACP (11.4 kDa); (6) *empty*; (8) vulcPPT (30.7 kDa); (9) Sfp R4-4 (27.3 kDa); (10) AcpS (16.2 kDa).

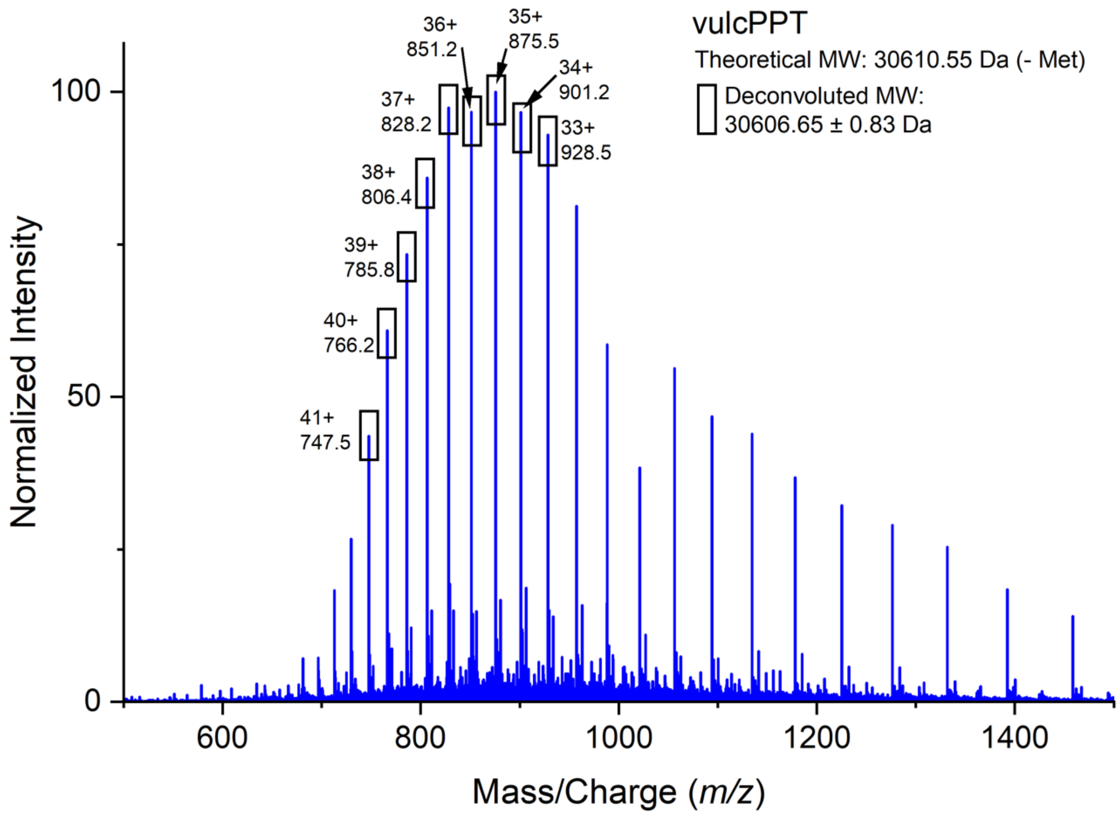

Figure S2. LC-MS spectrum of purified vulcPPT. The first nine major peaks were used for molecular weight deconvolution.

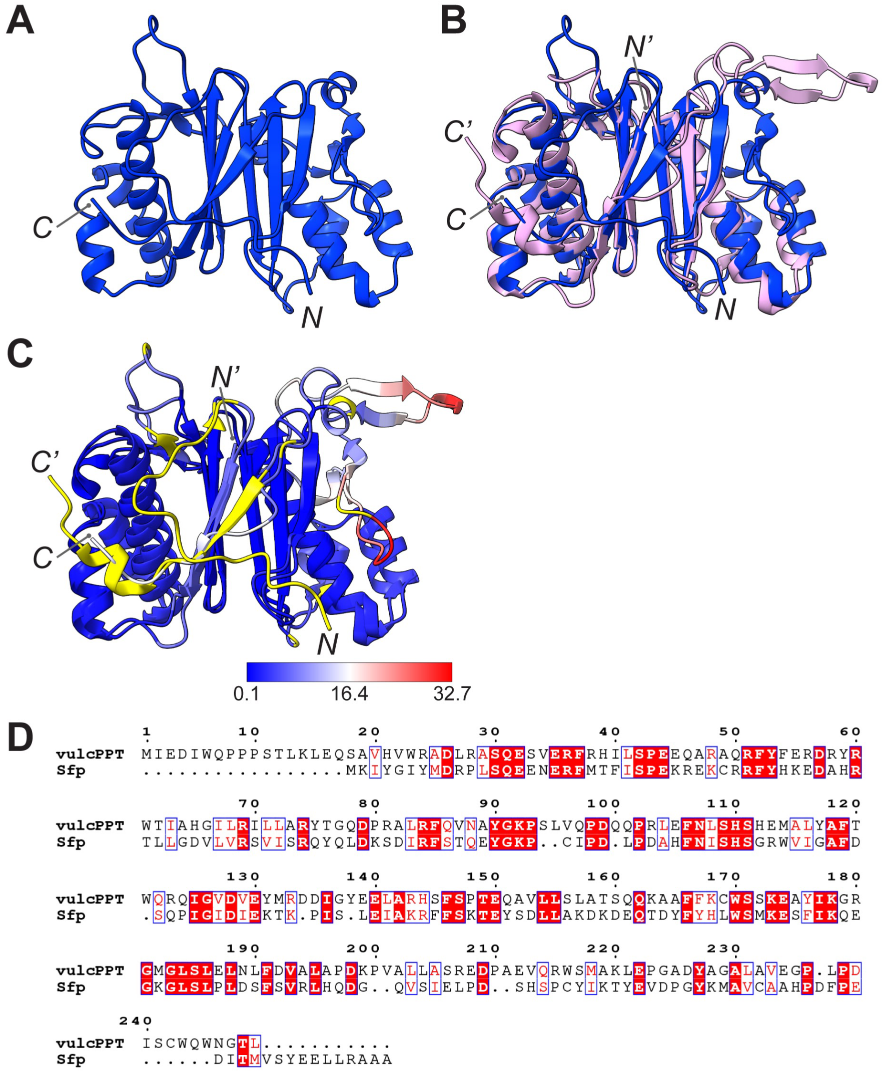

Figure S3. Structural comparison of vulcPPT and Sfp highlights their high structural similarity. (A) Predicted structure of vulcPPT generated using AlphaFold 3,[1] with the vulcPPT protein insert as input (see Table S1). The helicity was calculated to be 35.3%. (B) Structural overlay with Sfp (PDB 1QR0, pink) emphasizes their similarity. (C) The calculated RMSD between 127 pruned atom pairs is 1.089 Å. The color key indicates the sequence RMSD range from 0.1 Å (blue) to 32.7 Å (red), with unmatched residues shown in yellow. The *N*- and *C*-termini are labeled as *N* and *C* for vulcPPT, and *N'* and *C'* for Sfp. (D) Sequence alignment, performed using Clustal Omega[2] and ESPript 3,[3] indicates residue similarities as follows: red box with white character for strict identity, red character for group similarity, blue frame for similarity across groups, and black character for no similarity. The sequence from PDB 1QR0 (wild-type Sfp) was used for the alignment, while the R4-4 mutant of Sfp (Sfp R4-4)[4] was utilized in the experimental assays. The R4-4 mutant contains specific amino acid substitutions that enhance its substrate promiscuity compared to the wild-type enzyme: Lys28 (AAA) → Glu (GAG), Lys31 (AAA) → Lys (AAG), Thr44 (ACC) → Glu (GAG), His90 (CAC) → His (CAT), and Lys136 (AAG) → Lys (AAG).

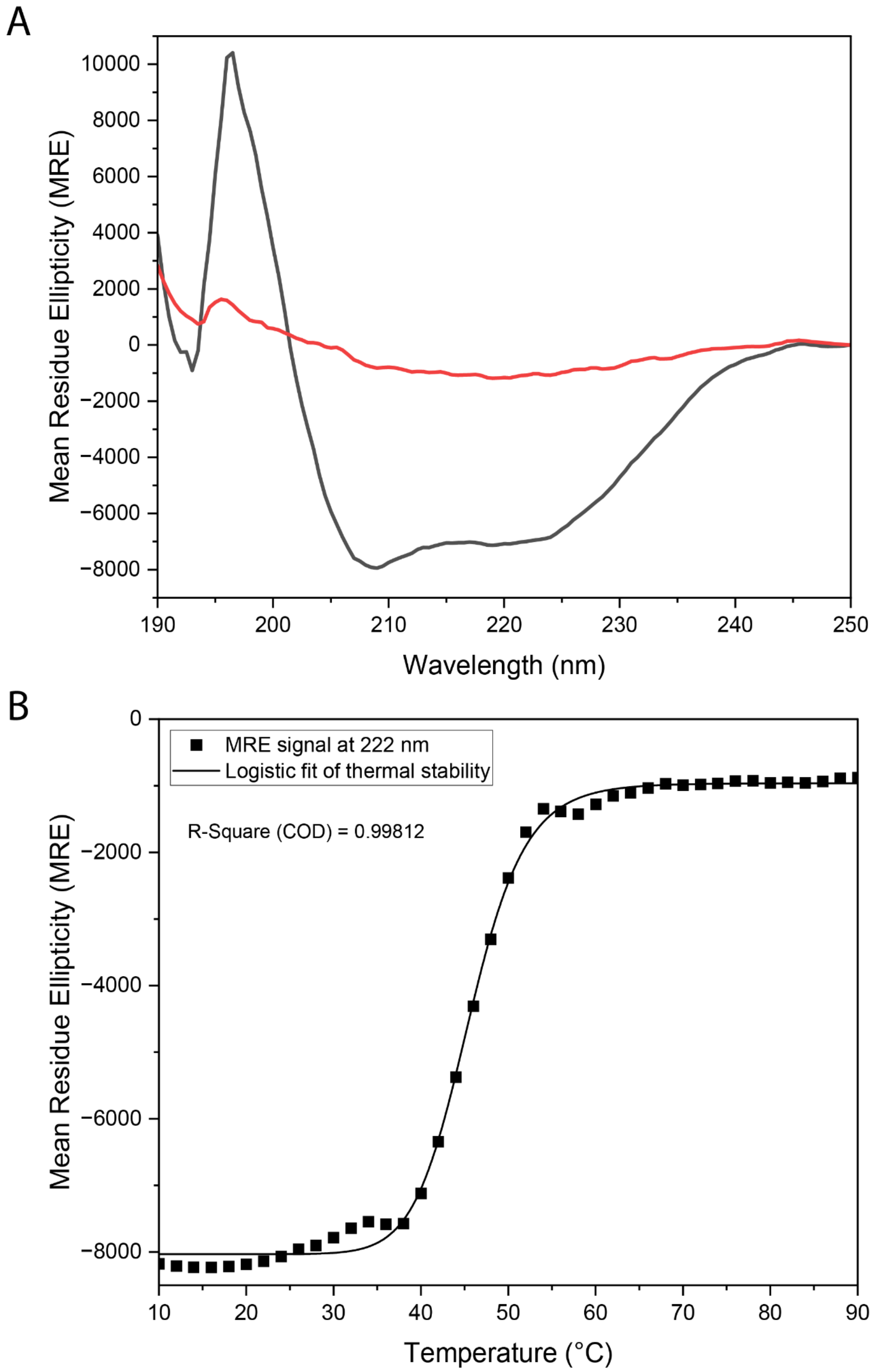

Figure S4. Circular dichroism spectrum and T_{melt} of vulcPPT. (A) Dark line represents the CD signal of folded vulcPPT in the far-UV range (190–250 nm) at 25 °C, prior to collecting the T_{melt} thermal stability curve of vulcPPT. Red line represents the CD signal in the far-UV range of denatured vulcPPT after completing the T_{melt} thermal stability curve of vulcPPT and cooling back down to 10 °C. VulcPPT does not refold after denaturation. (B) T_{melt} thermal stability curve of vulcPPT. CD signal at 222 nm was collected over a temperature range of 10–90 °C in 2 °C steps. All spectra were blank-corrected and a sigmoidal fit using a logistic function was fitted onto the data. E50/x0 value from the sigmoidal fit corresponded to a T_{melt} of 45.53 ± 0.149 °C. A second transition between 30–35 °C is possible, which could suggest a multi-domain structure or a unit of secondary structure that is partially stabilized through contact with the structure of the rest of the protein.

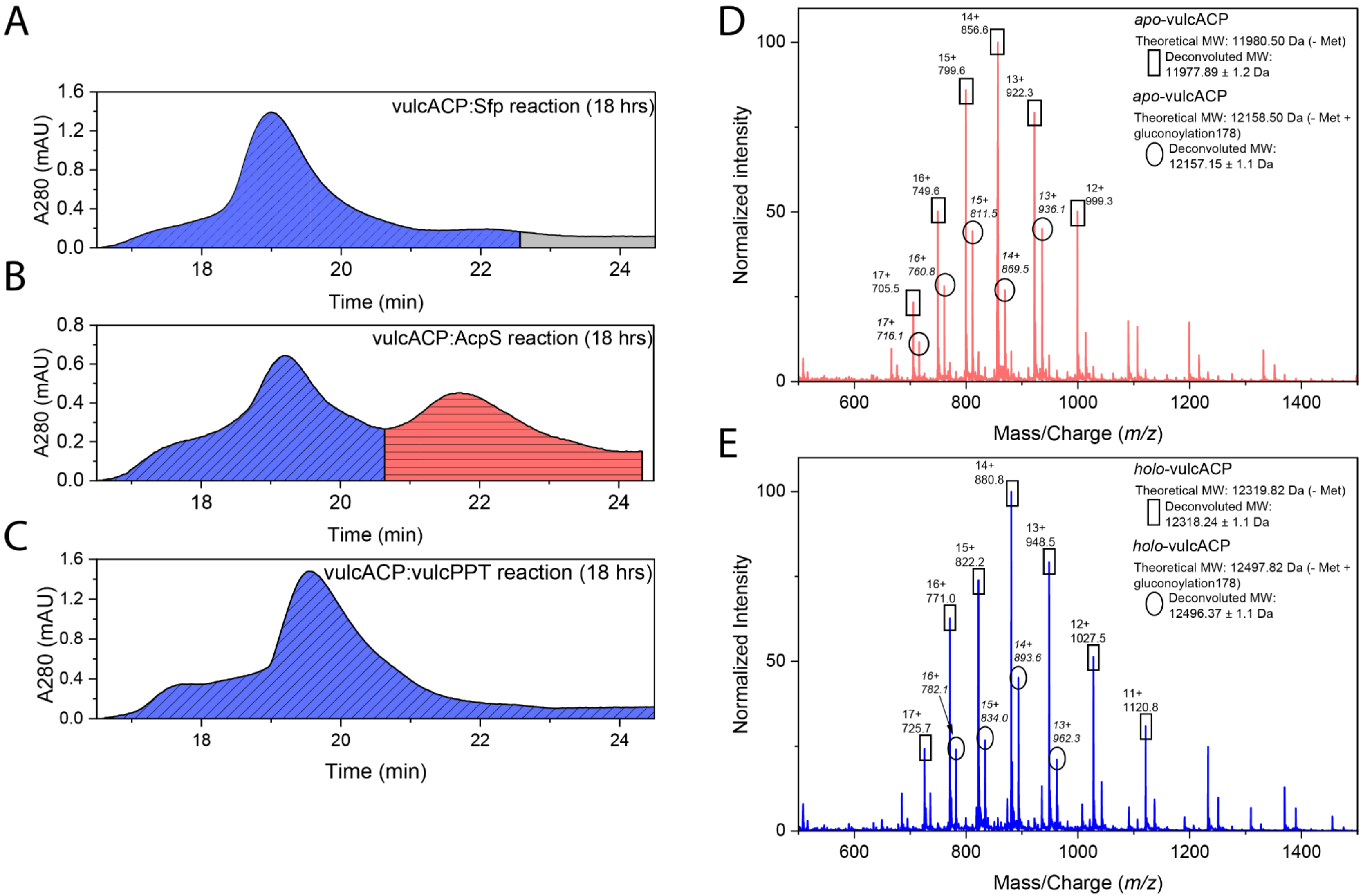

Figure S5. Mass spectra of *apo*- and *holo*-vulcACP, and LC chromatograms after PPTase reactions with Sfp, AcpS and vulcPPT. *Apo*- (red) and *holo*- (blue) ACP peaks were confirmed by mass-spectrometry. Areas without visible ACP are denoted in gray. (A) UV-Vis spectrum of the reaction between vulcACP and Sfp, showing full conversion to the *holo* form. (B) UV-Vis spectrum of the reaction between vulcACP and AcpS, showing an incomplete conversion. As a result, a mixture of *holo* and *apo* is observed. (C) UV-Vis spectrum of the reaction between vulcACP and vulcPPT, showing full conversion to the *holo* form. (D) Representative deconvoluted mass spectrum of *apo*-vulcACP. (E) Representative deconvoluted mass spectrum of *holo*-vulcACP.

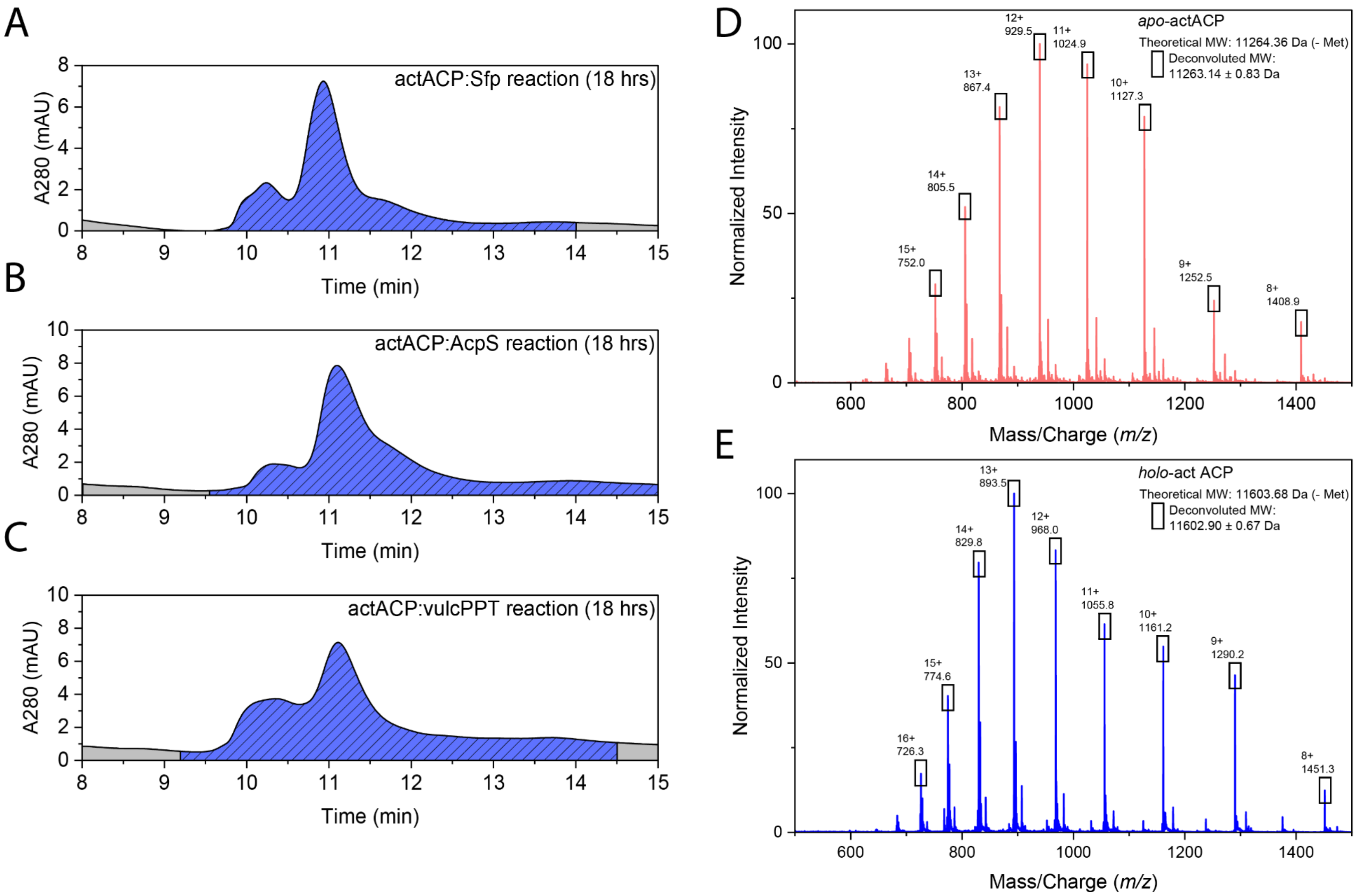

Figure S6. Mass spectra of *apo*- and *holo*-actACP, and LC chromatograms after PPTase reactions with Sfp, AcpS and vulcPPT. *Apo*- (red) and *holo*- (blue) ACP peaks were confirmed by mass-spectrometry. Areas without visible ACP are denoted in gray. (A) UV-Vis spectrum of the reaction between actACP and Sfp. (B) UV-Vis spectrum of the reaction between actACP and AcpS. (C) UV-Vis spectrum of the reaction between actACP and vulcPPT. All three PPTases show full conversion to the *holo* form. (D) Representative deconvoluted mass spectrum of *apo*-actACP. (E) Representative deconvoluted mass spectrum of *holo*-actACP.

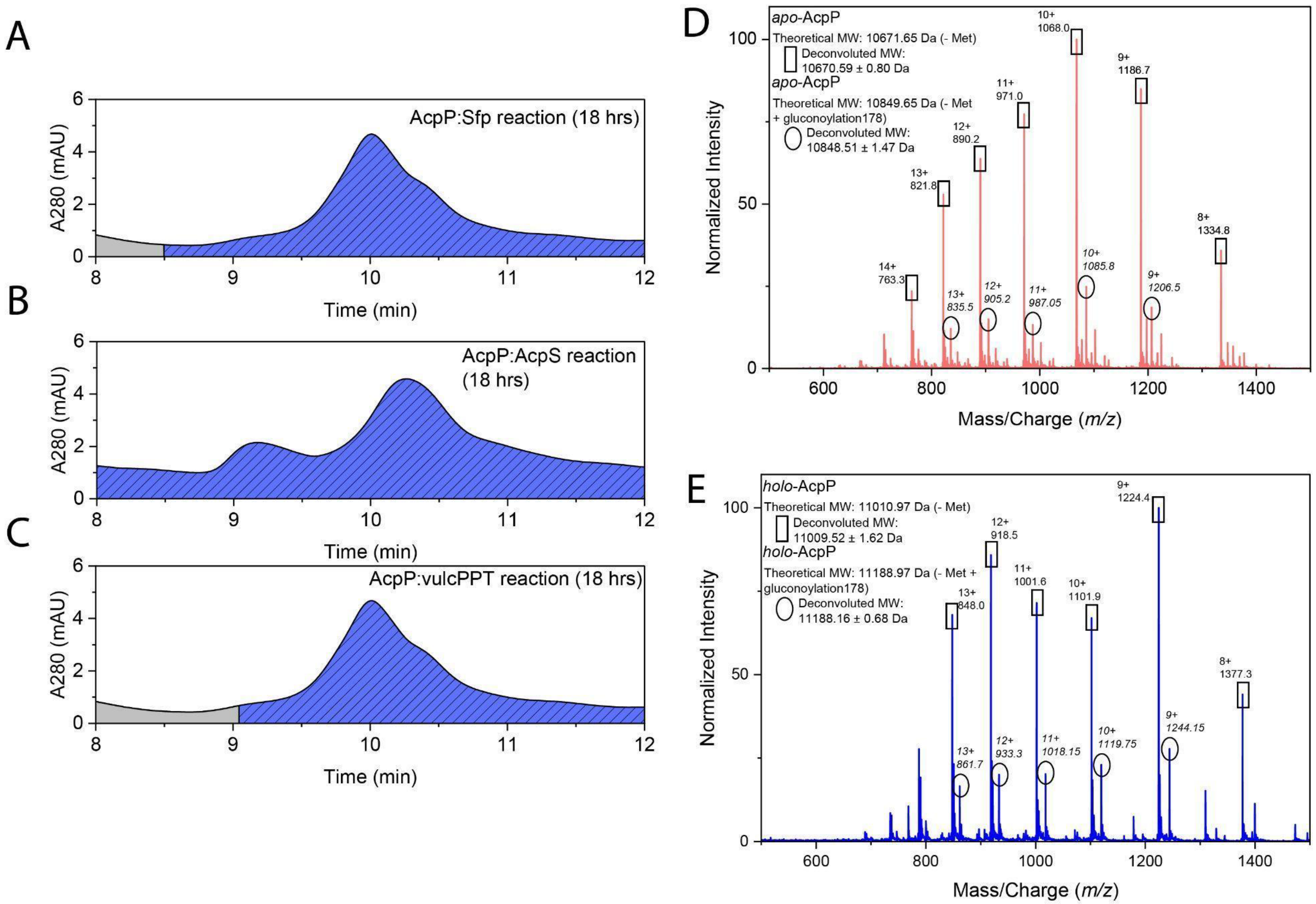

Figure S7. Mass spectra of *apo*- and *holo*-AcpP, and LC chromatograms after PPTase reactions with Sfp, AcpS and vulcPPT. *Apo*- (red) and *holo*- (blue) ACP peaks were confirmed by mass-spectrometry. Areas without visible ACP are denoted in gray. (A) UV-Vis spectrum of the reaction between AcpP and Sfp. (B) UV-Vis spectrum of the reaction between AcpP and AcpS. (C) UV-Vis spectrum of the reaction between AcpP and vulcPPT. All three PPTases show full conversion to the *holo* form. (D) Representative deconvoluted mass spectrum of *apo*-AcpP. (E) Representative deconvoluted mass spectrum of *holo*-AcpP.

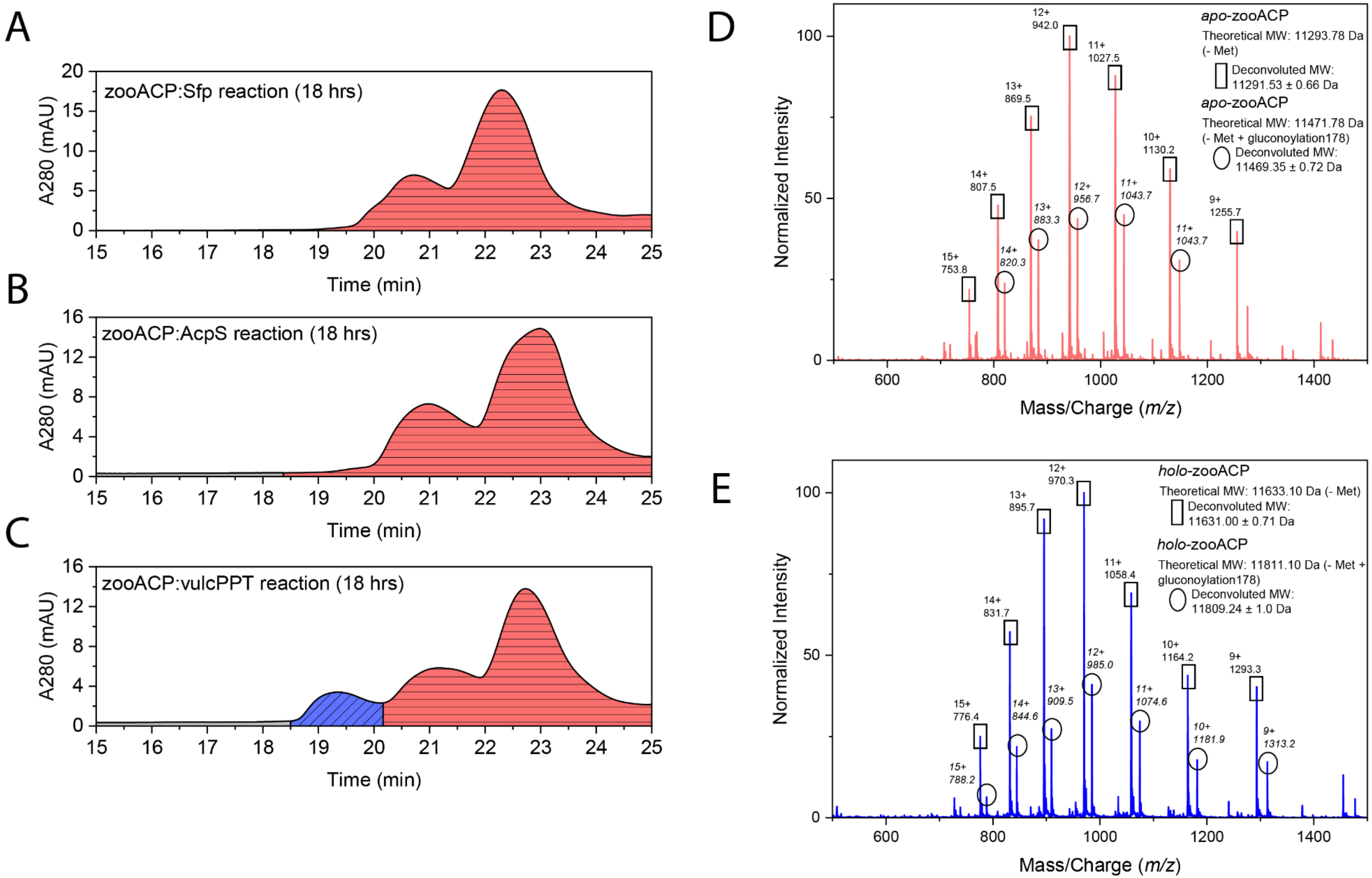

Figure S8. Mass spectra of *apo*- and *holo*-zooACP, and LC chromatograms after PPTase reactions with Sfp, AcpS and vulcPPT. *Apo*- (red) and *holo*- (blue) ACP peaks were confirmed by mass-spectrometry. Areas without visible ACP are denoted in gray. (A) UV-Vis spectrum of the reaction between zooACP and Sfp, showing no conversion to the *holo* form. (B) UV-Vis spectrum of the reaction between zooACP and AcpS, showing no conversion to the *holo* form. (C) UV-Vis spectrum of the reaction between zooACP and vulcPPT, showing an incomplete conversion to the *holo* form. As a result, a mixture of *holo* and *apo* is observed. (D) Representative deconvoluted mass spectrum of *apo*-zooACP. (E) Representative deconvoluted mass spectrum of *holo*-zooACP

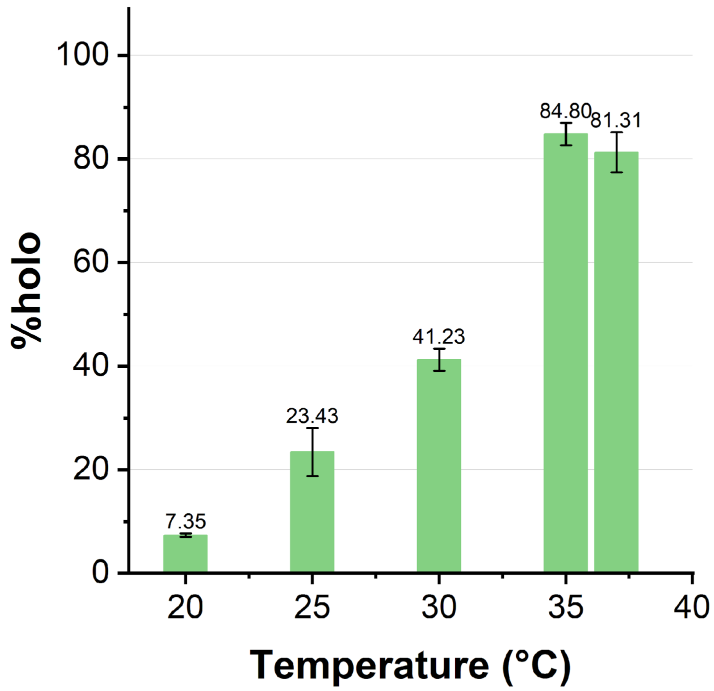

Figure S9. Temperature optimization of the phosphopantetheinylation of *apo*-zooACP by vulcPPT (detailed version of Fig 3A) at pH 7.6, demonstrating that 35 °C results in the highest conversion (84%) to the *holo* form.

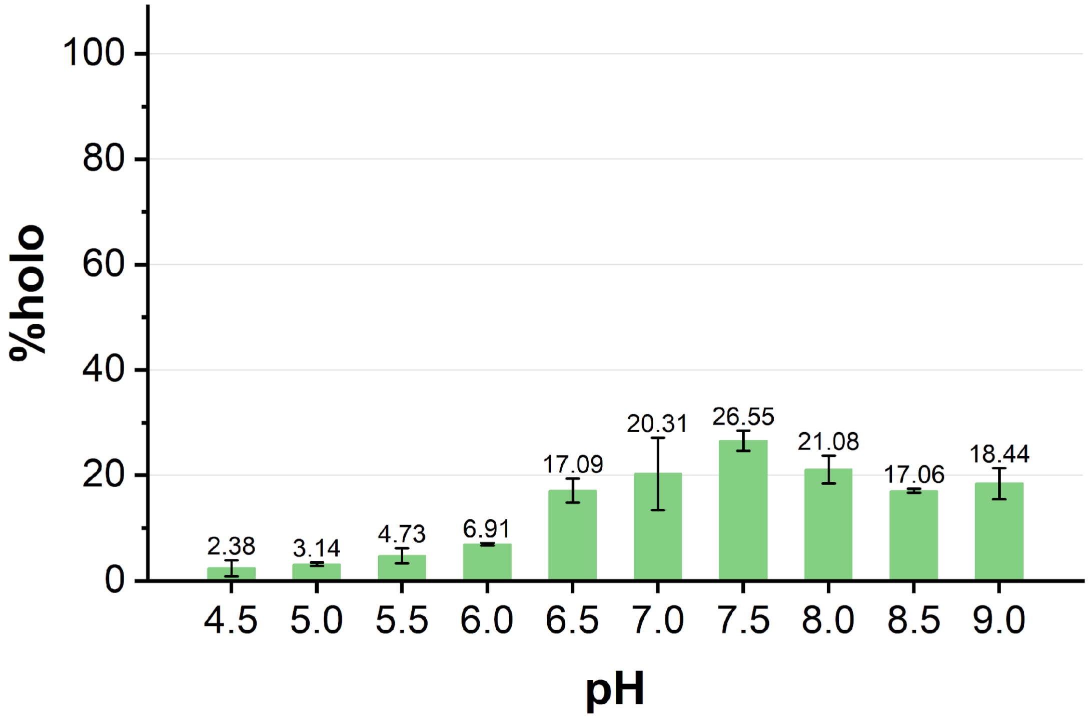

Figure S10. pH optimization of the phosphopantetheinylation of *apo*-zooACP by vulcPPT (detailed version of Fig 3B) at 22 °C, demonstrating that pH 7.5 results in the highest conversion (27%) to the *holo* form.

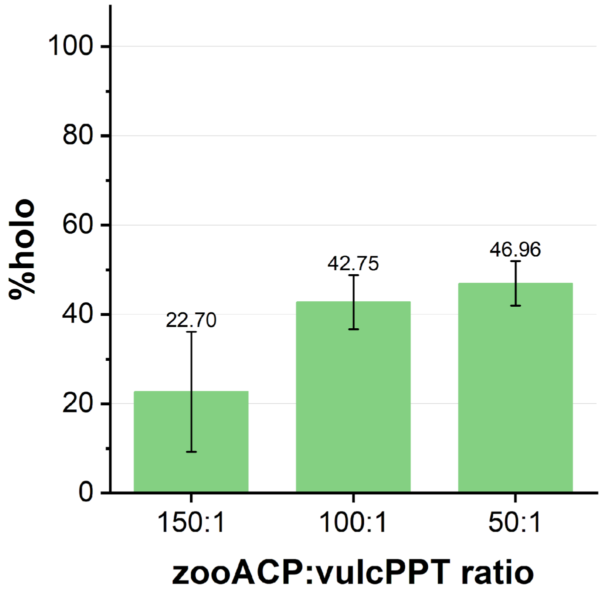

Figure S11. VulcPPT concentration optimization of the phosphopantetheinylation of *apo*-zooACP by vulcPPT. Three ratios of differing molar concentrations were tested: 150:1 (150 µM ACP to 1.0 µM vulcPPT), 100:1 (150 µM ACP to 1.5 µM vulcPPT) and 50:1 (150 µM ACP to 3.0 µM vulcPPT). The 50:1 zooACP:vulcPPT ratio showed the highest conversion to the *holo* form (47%), although within the standard deviation of the 100:1 ratio.

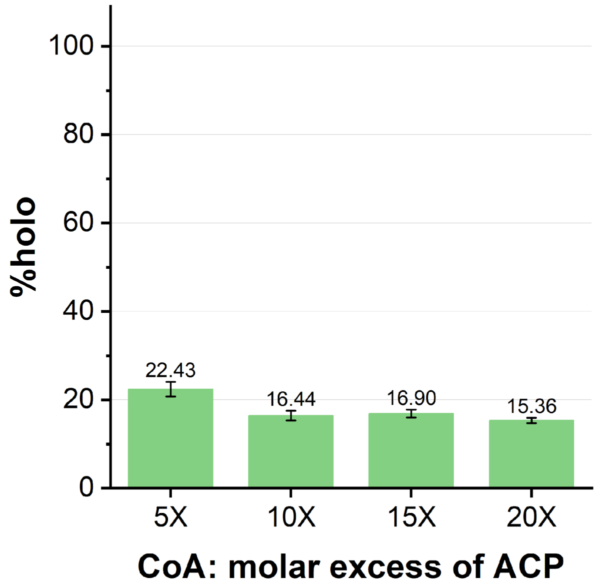

Figure S12. Coenzyme A concentration optimization of the phosphopantetheinylation of *apo*-zooACP by vulcPPT. Four CoA concentrations were tested at molar excesses of 5x (750 µM), 10x (1.50 mM), 15x (2.25 mM), and 20x (3.00 mM) relative to 150 µM *apo*-zooACP. The 5x CoA concentration resulted in the highest conversion to the *holo* form (22%), though the significance was not major compared to the other CoA concentrations.

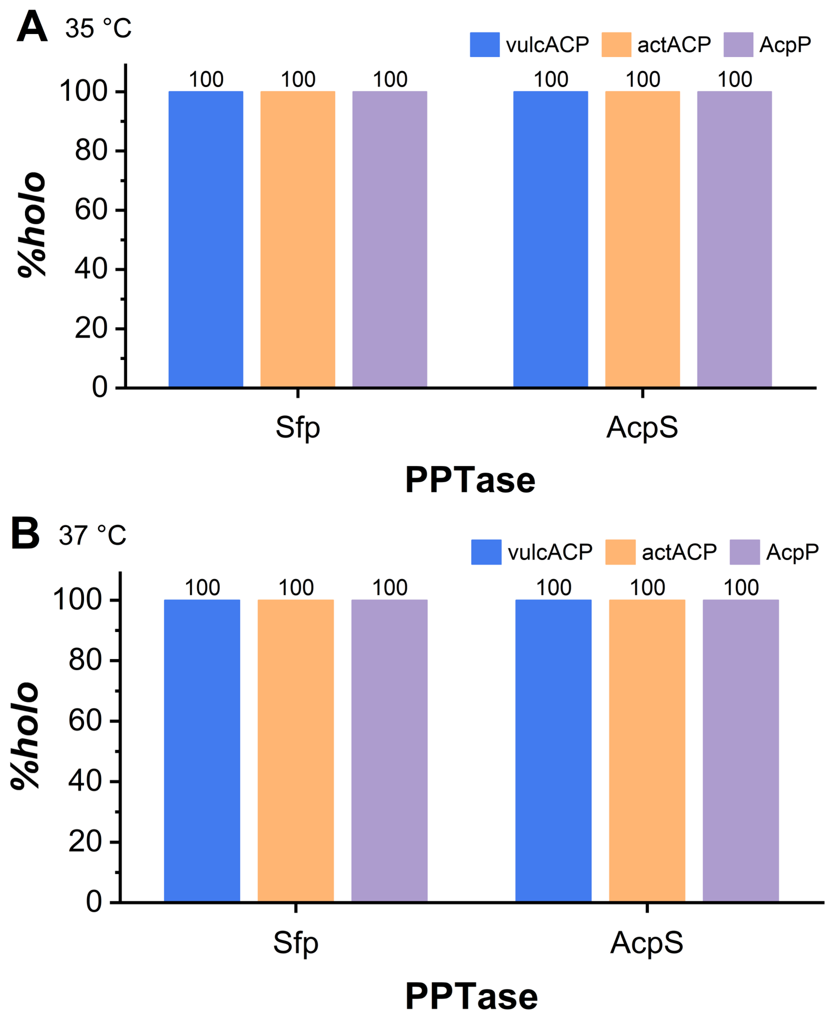

Figure S13. Phosphopantetheinylation reactions of Sfp and AcpS with vulcACP, actACP, and AcpP at 35 ºC and 37 ºC. When *apo*-vulcACP (blue), *apo*-actACP (orange), and *apo*-AcpP (purple) were reacted with Sfp, AcpS, and vulcPPT (see Materials and Methods for reaction conditions) for 18 hrs at 35 ºC and 37 ºC all ACPs were found to be in 100% *holo*-state via LC-MS.

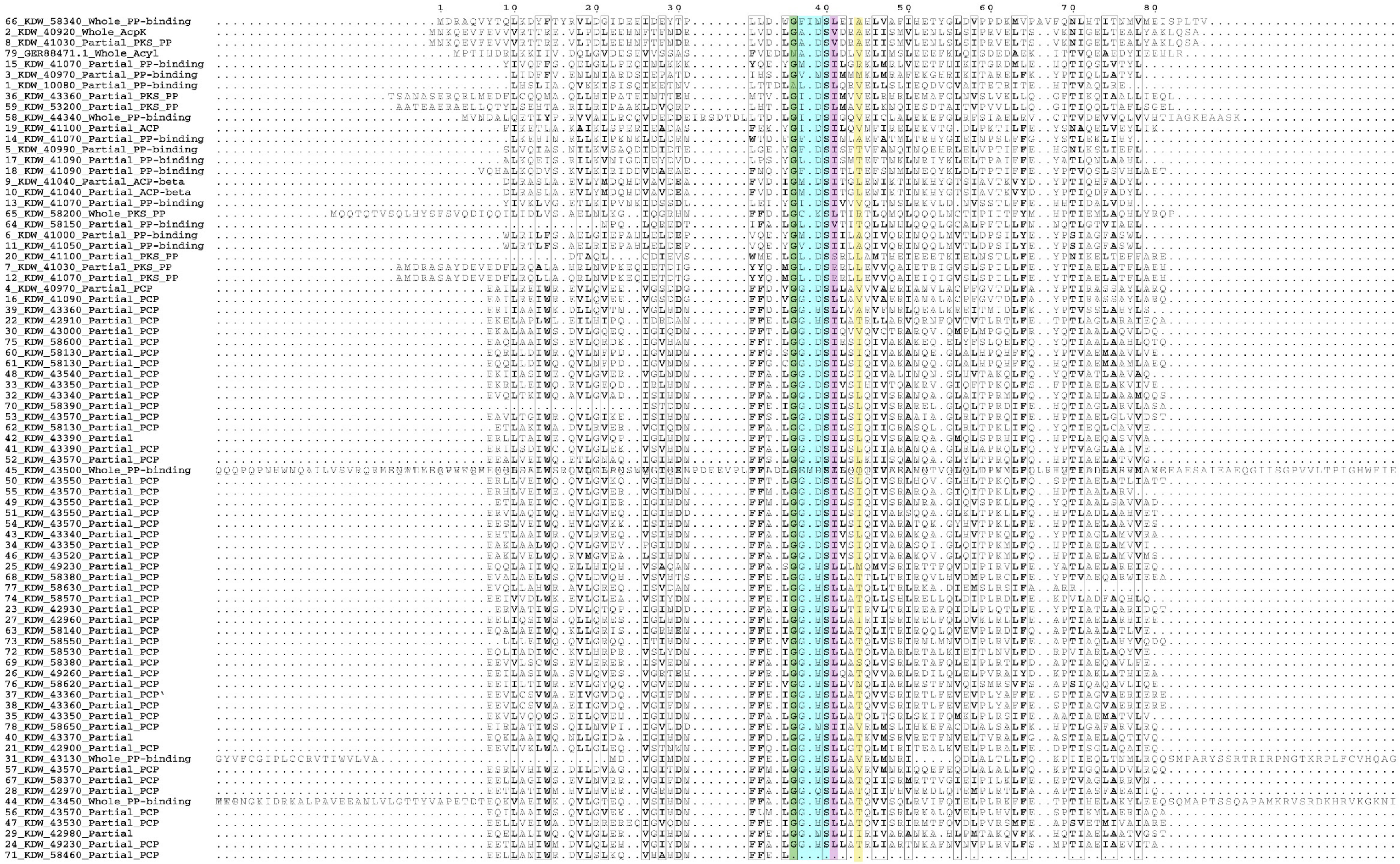

Figure S14. Multiple sequence alignment of 79 carrier proteins encoded by the *Dictyobacter vulcani* sp. W12 genome. 97.5% (77/79) of the CPs have a G as the first residue of the motif. Only 6.3% of the CPs shared the traditional Sfp-favored sequence “DSL”, instead having DSI (44.3%, 35/79), HSL (34.1%, 27/79), or some other amino acid triad in its place.

References for Supporting Information

(1) Abramson, J.; Adler, J.; Dunger, J.; Evans, R.; Green, T.; Pritzel, A.; Ronneberger, O.; Willmore, L.; Ballard, A. J.; Bambrick, J.; Bodenstein, S. W.; Evans, D. A.; Hung, C.-C.; O'Neill, M.; Reiman, D.; Tunyasuvunakool, K.; Wu, Z.; Žemgulytė, A.; Arvaniti, E.; Beattie, C.; Bertolli, O.; Bridgland, A.; Cherepanov, A.; Congreve, M.; Cowen-Rivers, A. I.; Cowie, A.; Figurnov, M.; Fuchs, F. B.; Gladman, H.; Jain, R.; Khan, Y. A.; Low, C. M. R.; Perlin, K.; Potapenko, A.; Savy, P.; Singh, S.; Stecula, A.; Thillaisundaram, A.; Tong, C.; Yakneen, S.; Zhong, E. D.; Zielinski, M.; Žídek, A.; Bapst, V.; Kohli, P.; Jaderberg, M.; Hassabis, D.; Jumper, J. M. Accurate Structure Prediction of Biomolecular Interactions with AlphaFold 3. *Nature* **2024**, *630*, 493.

(2) Sievers, F.; Higgins, D. G. Clustal Omega. *Curr. Protoc. Bioinform.* **2014**, *48,* 3.1.1.

(3) Winkler, R. ESIprot: A Universal Tool for Charge State Determination and Molecular Weight Calculation of Proteins from Electrospray Ionization Mass Spectrometry Data. *Rapid Commun. Mass Spectrom.* **2010**, *24*, 285.

(4) Sunbul, M.; Marshall, N. J.; Zou, Y.; Zhang, K.; Yin, J. Catalytic Turnover-Based Phage Selection for Engineering the Substrate Specificity of Sfp Phosphopantetheinyl Transferase. *J. Mol. Biol.* **2009**, *387*, 883.
